# The Role of Sirtuin1 in Regulating Endothelial Function, Arterial Remodeling and Vascular Aging

**DOI:** 10.3389/fphys.2019.01173

**Published:** 2019-09-12

**Authors:** Andy W. C. Man, Huige Li, Ning Xia

**Affiliations:** Department of Pharmacology, University Medical Centre, Johannes Gutenberg University Mainz, Mainz, Germany

**Keywords:** eNOS, SIRT1, PVAT, vascular remodeling, vascular aging

## Abstract

Sirtuin1 (SIRT1), which belongs to a highly conserved family of protein deacetylase, is one of the best-studied sirtuins. SIRT1 is involved in a variety of biological processes, including energy metabolism, cell proliferation and survival, chromatin dynamics, and DNA repair. In the vasculature, SIRT1 is ubiquitously expressed in endothelial cells, smooth muscle cells, and perivascular adipose tissues (PVAT). Endothelial SIRT1 plays a unique role in vasoprotection by regulating a large variety of proteins, including endothelial nitric oxide synthase (eNOS). In endothelial cells, SIRT1 and eNOS regulate each other synergistically through positive feedback mechanisms for the maintenance of endothelial function. Recent studies have shown that SIRT1 plays a vital role in modulating PVAT function, arterial remodeling, and vascular aging. In the present article, we summarize recent findings, review the molecular mechanisms and the potential of SIRT1 as a therapeutic target for the treatment of vascular diseases, and discuss future research directions.

## Introduction

Sirtuins are a highly conserved family of nicotinamide adenine dinucleotide (NAD^+^)-dependent protein deacetylase that regulate various signaling molecules, including transcription factors, histones, and enzymes (Satoh et al., 2011). In mammals, the sirtuin family consists of seven members, SIRT1-7, sub-divided into classes I-VI. Most of the sirtuin family members are considered to be potential regulators of metabolism and aging (Yamamoto et al., 2007).

Sirtuin1 (SIRT1) is the most studied member of the sirtuin family and is known to shuttle between the nucleus and cytoplasm (Tanno et al., 2007). SIRT1 is involved in a variety of biological processes, including energy metabolism, cell proliferation and survival, chromatin dynamics, and DNA repairment (Wood et al., 2004; Donmez and Guarente, 2010), as also demonstrated by the shortened life span of SIRT1 knockout mice compared to wild type mice (Li et al., 2008). SIRT1 expressed in endothelial cells plays a unique role in vasoprotection by regulating a variety of substrates that include endothelial nitric oxide synthase (eNOS), liver kinase B1 (LKB1), and forkhead box O1 (FOXO1) (Zu et al., 2010; Xia et al., 2013; Bai et al., 2014), and exerts its vasoprotective effects by preventing endothelial senescence, promoting endothelial angiogenesis and migration, enhancing endothelium-dependent vasodilatation, and suppressing vascular inflammation and foam cell formation (Stein and Matter, 2011; Bai et al., 2014). A reciprocal regulation or synergism between SIRT1- and eNOS-mediated signaling pathways have also been reported in promoting endothelial functions (Mattagajasingh et al., 2007). In addition to the improvement of endothelial function, the vasoprotective effects of SIRT1 in preventing perivascular adipose tissue (PVAT) dysfunction and adverse arterial remodeling are critical to the cardiovascular system, as evidenced by recent studies (Xia and Li, 2017). In this review, we discuss the relationship between SIRT1 and eNOS and the novel roles of SIRT1 in PVAT function and arterial remodeling and aging.

## Interplay Between SIRT1 and eNOS in Regulating Endothelial Function

Endogenous nitric oxide (NO) generated from eNOS plays a crucial role in maintaining endothelial function and homeostasis and regulates vascular tone, leukocyte adhesion, smooth muscle cell proliferation and migration, and platelet aggregation (Guo and Wang, 2018). eNOS also contributes to oxidative stress resistance by producing NO and inhibiting O_2_^–^ generation (Förstermann and Sessa, 2011). In the endothelium, SIRT1 influences the regulation of transcription and enzymatic activity of eNOS, leading to enhanced NO production (Wallerath et al., 2002; Xia et al., 2013). The interplay between SIRT1 and eNOS in regulating endothelial function and defending oxidative stress has been reported extensively.

Resveratrol is a plant polyphenol that activates SIRT1 (Xia et al., 2017a). Interestingly, resveratrol treatment in endothelial cells leads to upregulation of eNOS expression (Wallerath et al., 2002). The effect of resveratrol on eNOS expression is independent of the estrogen receptor (Wallerath et al., 2002) but mediated by SIRT1. In endothelial cells, the knock-down of SIRT1 gene via siRNA inhibits the upregulation of eNOS by resveratrol treatment (Csiszar et al., 2009). Consistently, an endothelium-specific overexpression of SIRT1 leads to elevation of eNOS expression (Zhang et al., 2008). Therefore, resveratrol-induced upregulation of eNOS is likely to be SIRT1-dependent. To strengthen this hypothesis, we have also shown that the SIRT1/FOXO factor axis is critically involved in resveratrol-induced eNOS transcriptional activation (Xia et al., 2013). Knock-down of FOXO1 and FOXO3a blocks the resveratrol-induced eNOS transcriptional activation in human endothelial cells (Xia et al., 2013). These findings suggest that FOXO factors are the downstream targets of SIRT1 in mediating the eNOS expressional regulation by resveratrol. In addition to SIRT1/FOXO, eNOS and NO are also induced in a SIRT1/Krüpple link factor 2 (KLF2)-dependent manner and regulate endothelial function (Cui et al., 2012). Another SIRT1 activator, SRT1720, is also shown to increase eNOS expression, and possess anti-oxidative and anti-inflammatory action via NF-κB and AMPK-dependent mechanisms in rats (Wang et al., 2019), confirming an important relationship between SIRT and eNOS expression.

In addition to eNOS expression, SIRT1 also increases eNOS enzymatic activity by deacetylation. In endothelium, SIRT1 is physically associated with eNOS. Knock-down of SIRT1 or inhibition of SIRT1 activity enhances eNOS acetylation on lysine 496 and 506 residues in the calmodulin-binding domain (Mattagajasingh et al., 2007). In contrast, eNOS acetylation is decreased by SIRT1 overexpression or stimulation of SIRT1 activity by resveratrol (Mattagajasingh et al., 2007). Adenovirus-mediated overexpression of the dominant-negative SIRT1 mutant impairs acetylcholine-induced NO production and endothelium-dependent relaxation in rat aorta (Mattagajasingh et al., 2007). Thus, SIRT1 regulates both eNOS expression and activity.

Conversely, eNOS-derived NO also regulates SIRT1 expression, thereby establishing a partnership between SIRT1 and eNOS. SIRT1 expression is regulated by NO in white adipose tissue (WAT) and white adipocytes (Nisoli et al., 2005). Calorie restriction leads to enhanced SIRT1 expression in WAT of wild-type mice but not in eNOS knockout mice. In cultured adipocyte, the SIRT1 expression is enhanced by treatments of NO donors and cGMP analogs (Nisoli et al., 2005). SIRT1 expression is also upregulated by overexpression of eNOS in the cultured mouse pancreatic β-cell line Min6 (Hu et al., 2017). These indicate the importance of eNOS-derived NO as a regulator of SIRT1 expression. Moreover, resveratrol treatment also mimics the effect of short-term calorie restriction in cardiovascular protection, including the reduction of several stress response pathways (Barger et al., 2008). Long-term resveratrol treatment in mice can mimic the transcriptional activation induced by dietary restriction and promote healthy aging by improving vascular function (Pearson et al., 2008). The above studies indicate that resveratrol may induce calorie restriction-mimicking effects without affecting SIRT1 expression, but an increased level of eNOS mRNA expression is detected. These suggest the vital role of resveratrol in vascular health in regulating eNOS expression but not directly via SIRT1 induction.

**FIGURE 1:**
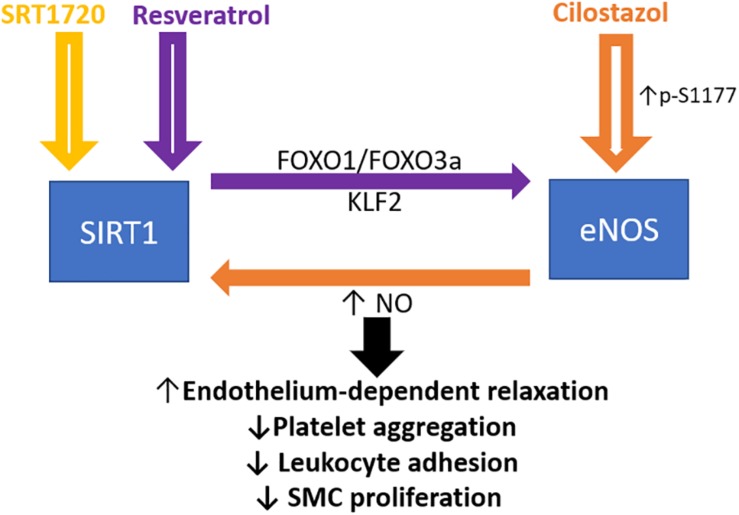
Crosstalk between endothelial SIRT1 and eNOS in mediating endothelial function. Schematic presentation of the current idea on the reciprocal regulation of SIRT1 and eNOS. Resveratrol activates both SIRT1 and eNOS, whereas FOXO1 and FOXO3a are involved in the resveratrol induced eNOS transcriptional activation, based on the published literature (Xia et al., 2013). Cilostazol increases NO production by stimulating eNOS serine 1177 phosphorylation mediated by cAMP/PKA- and PI3K/Akt-dependent pathways. NO, nitric oxide; FOXO, forkhead box O; SMC, smooth muscle cells. KLF2, Krüppel-like Factor 2.

In the endothelial cell, eNOS-derived NO also regulates SIRT1 expression during aging. Uncoupling of eNOS decreases the expression of endothelial SIRT1 (Lemarié et al., 2011). The phosphodiesterase enzyme 3 (PDE3) inhibitor cilostazol prevents premature endothelial senescence by a NO-dependent upregulation of SIRT1 (Ota et al., 2008). Cilostazol increases NO production by stimulating eNOS serine 1177 phosphorylation mediated by cAMP/PKA- and PI3K/Akt-dependent pathways. The cilostazol-induced SIRT1 expression can be blocked by NOS inhibitor L-NAME, indicating the role of eNOS-derived NO in SIRT1 upregulation (Ota et al., 2010). Therefore, a positive feedback loop exists in the eNOS-NO-SIRT1 axis to protect against endothelial dysfunction, senescence, and atherosclerosis (Figure 1). The interplay between eNOS and SIRT1 under pathophysiological conditions, including vascular remodeling and aging, will be further discussed.

## Role of SIRT1 and eNOS in Arterial Remodeling

Arterial remodeling is the active process of structural alteration that occurs as a result of cell death, proliferation, and migration as well as changes in the extracellular matrix of a vessel (Man and Wang, 2017), and is controlled by the crosstalk between endothelium and vascular smooth muscle cells. Endothelial cells can sense shear stress from blood flow and activate signaling pathways in vascular smooth muscle cells (Man and Wang, 2017). Several factors are involved in the extracellular matrix formation and remodeling, including collagen type I and III, macrophages, fibronectin, laminin, elastin, and proteoglycans (Lu et al., 2011); abnormal arterial remodeling contributes to the development of hypertension and other vascular disorders (Intengan and Schiffrin, 2001). Vasculature in hypertensive individuals undergoes accelerated vascular wall thickening, leading to degeneration and calcification of the vascular wall and increased vessel stiffness, while compensatory vessel wall enlargement is observed in atherosclerotic patients (Rudic and Sessa, 1999).

To date, there are limited reports concerning the role of endothelial SIRT1 in arterial remodeling. In clinical studies, the genetic variations of SIRT1 are found to be associated with the changes in intimal-medial thickness in human carotid arteries, suggesting that SIRT1 function is related to arterial remodeling (Kedenko et al., 2014). Coherent results are reported in studies using mouse arteries. Neointima formation in the blood vessel is associated with a progressive downregulation of SIRT1 expression, while the overexpression of SIRT1 in vascular smooth muscle cells represses neointima formation in response to vascular injury (Li et al., 2011). Furthermore, inhibition of SIRT1 increases the expression of p53 and its downstream signaling target, plasminogen activator inhibitor-1 (PAI-1), leading to the formation of neointima and vascular remodeling in response to vascular injury (Wan et al., 2014).

Resveratrol has been reported to prevent high-fat, high-sucrose diet (HFHS)-induced arterial stiffening in mice (Fry et al., 2016). Similar results obtained from experiments with SIRT1 activators or SIRT1 overexpression indicate that the beneficial effect of resveratrol treatment is likely mediated by SIRT1 (Fry et al., 2016). Overnight fasting acutely decreases arterial stiffness in control mice, but not in mice lacking SIRT1 in vascular smooth muscle cells. Conversely, vascular smooth muscle cells-specific SIRT1 overexpression prevents diet-induced arterial stiffening. The anti-stiffening effect of SIRT1 has been attributed to its anti- antioxidant and inflammatory properties mediated by inhibition of nuclear factor kappa-light-chain-enhancer of activated B cells (NF-κB) and downregulation of vascular cell adhesion protein 1 (VCAM-1) and p47phox (Fry et al., 2016).

Conversely, SIRT1 overexpression in the smooth muscle cells prevents angiotensin II (AngII)-induced activation of matrix metalloproteinase (MMP) and neointimal remodeling (Wan et al., 2014). In cultured endothelial cells, laminar flow increases both SIRT1 expression and activity, whereas oscillating flow decreases the expression of SIRT1, suggesting that disturbances of the local blood flow in many vascular diseases contribute to arterial remodeling by downregulating SIRT1 (Chen et al., 2010; Yao et al., 2013). Resveratrol downregulates AngII type 1 receptor expression in vascular smooth muscle cells through SIRT1 activation both *in vivo* and *in vitro* (Miyazaki et al., 2008). Resveratrol treatment also leads to the reduction in serum AngII level and expression of prorenin receptor (PRR) and angiotensin-converting enzyme (ACE), ACE2, AngII type 2 receptor (AT2R), and Mas receptor (MasR) in the aorta. Resveratrol is also found to normalize the AngI and II level, ACE, AT2R, and MasR expression through restoring SIRT1 level in HFD-mice (Sheen et al., 2018) and HFD-rats (Tiao et al., 2018). The protective effects of resveratrol on aging-induced vascular fibrosis may be mediated by SIRT1, which also modulates the renin-angiotensin system (Jang et al., 2018).

Nitric oxide derived from eNOS is an important endothelial regulator of flow- and pressure-induced arterial remodeling, and NO deficiency leads to endothelial dysfunction and abnormal vascular remodeling (Rudic and Sessa, 1999). The congenital absence of eNOS causes adverse vascular remodeling (Ozaki et al., 2001; Kobs and Chesler, 2006). NO produced by eNOS promotes smooth muscle relaxation and inhibits adverse arterial remodeling (Csiszar et al., 2002; Hussein et al., 2014). Long term treatment of L-NAME in Wistar-Kyoto rats causes a substantial increase in systemic blood pressure and microvascular remodeling, indicated by the increase in wall-to-lumen ratios and perivascular fibrosis (Numaguchi et al., 1995). The media layer of the abnormally remodeled vessels from eNOS knockout mice shows hyperplasia, as evidenced by the significant increase in wall thickness, number of medial nuclei, and the incorporation of bromodeoxyuridine, which reminisce the arterial thickening in hypertension and atherosclerosis of human patients (Moroi et al., 1998). Abnormal flow-dependent remodeling in eNOS knockout mice is associated with activation of the platelet-derived growth factor (PDGF) signaling pathway (Yu et al., 2012). In eNOS-knockout mice, the nuclear accumulation of acetylated LKB1 stimulates transforming growth factor beta (TGFβ)-mediated smooth muscle cells activation and collagen deposition in the vessel, leading to irreversible adverse arterial remodeling and vascular stiffening (Man et al., 2016). However, no detailed mechanisms are clarified on how eNOS depletion leads to the downstream pathways to mediate vascular remodeling.

**FIGURE 2:**
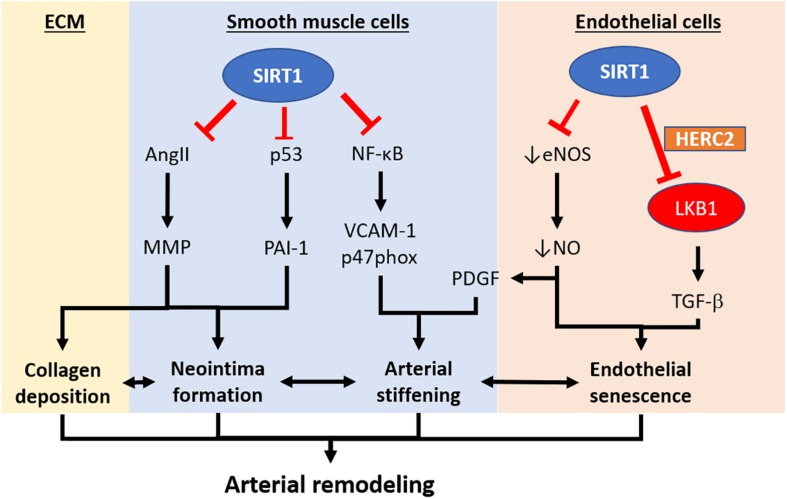
Pathways implicating the development of arterial remodeling and the role of SIRT1 in targeting arterial remodeling. Arterial remodeling is the structural alteration of arteries resulting from endothelial senescence/dysfunction, SMC activation, and changes in the extracellular matrix. Based on the current limited literature, SIRT1 in vascular smooth muscle cells is responsible for repressing neointima formation via a p53-PAI-1 pathway and inhibits Ang-II induced remodeling and intimal thickening. SIRT1 in vascular smooth muscle cells also prevents arterial stiffening via inhibition of NF-κB and downregulation VCAM-1 and p47phox. Endothelial SIRT1 facilitates protein complex formation with HERC2 and LKB1, which in turn, enhances LKB1 degradation and downregulation of TGFβ-signaling. SIRT1-HERC2-LKB1 axis prevents arterial remodeling by targeting endothelial senescence and SMC activation. NF-κB, nuclear factor kappa-light-chain-enhancer of activated B cells. VCAM-1, vascular cell adhesion protein 1. AngII, angiotensin II. MMP, matrix metalloproteinase. PAI-1, plasminogen activator inhibitor-1. TGFβ, transforming growth factor-beta. NO, nitric oxide. PDGF, platelet-derived growth factor. HERC2, HECT and RLD domain containing E3 ubiquitin-protein ligase 2.

Other than its function as a deacetylase, endothelial SIRT1 is also recently reported to facilitate protein complex formation and mediate protein degradation (Figure 2). The serine/threonine-protein kinase LKB1 plays an important role in endothelial senescence and vasculogenesis/arteriogenesis (Zu et al., 2010). SIRT1 prevents adverse arterial remodeling by enhancing LKB1 degradation via promoting the protein complex formation with HECT and RLD domain containing E3 ubiquitin-protein ligase 2 (HERC2), a giant scaffolding E3 ubiquitin ligase, but not by its deacetylase activity (Man et al., 2016). The reduced LKB1 in the endothelial cell results in the downregulation of TGFβ-mediated smooth muscle cells activation and collagen deposition in the vessel. Man et al. (2016) suggest that the SIRT1-HERC2-LKB1 protein complex formation is important in regulating LKB1 level and regulates arterial remodeling. Therefore, endothelial SIRT1 actions are not only limited to its deacetylase properties, but also to events related to protein complex formation and proteasome-mediated degradation. The proposed SIRT1-HERC-LKB1 pathway in targeting arterial remodeling is likely to be eNOS-independent (Man et al., 2016). However, SIRT1 and eNOS partner in preventing adverse vascular remodeling remain elusive.

## Association of SIRT1 AND ENOS IN Modulating PVAT Functions

Perivascular adipose tissue is the adipose tissue that surrounds large arteries and veins, small and resistance vessels, and skeletal muscle microvessels (Xia and Li, 2017). Recently, PVAT has been discovered as an important player in vascular biology. The contribution of PVAT in vascular function was first described by the observation that PVAT diminishes agonists−induced contractile responses in obese rat aortae *in vitro* (Soltis and Cassis, 1991). It is proposed that factors released by the PVAT reach the medial and endothelial layer of blood vessels either by direct diffusion or via the vasa vasorum (Gil-Ortega et al., 2015). It is known that PVAT regulates vascular function via endocrine or paracrine mechanisms by releasing various factors, including adipokines, cytokines/chemokines, and reactive oxygen species (Xia and Li, 2017). Dysfunction of PVAT may lead to complications in vascular functions and remodeling.

The primary mechanism leading to PVAT dysfunction, include aging, an increase in oxidative stress and inflammatory response, remodeling of PVAT (increase in adiposity and leptin/adiponectin dysregulation), and the loss of eNOS and NO (Fernández-Alfonso et al., 2013), leading to the imbalance of PVAT-derived adipokines, and affecting the vascular function. Our previous studies suggest that not obesity itself, but the dysfunction of PVAT, is responsible for the obesity-induced vascular disorder. When the PVAT is intact due to eNOS dysfunction, we have observed a significant reduction in the vasodilator response to acetylcholine in the aorta rings of obese mice compared to lean mice *in vitro* (Xia et al., 2016). The observed eNOS dysfunction in the PVAT is caused by the deficiency of L-arginine due to arginase induction; and Akt inhibition mediated reduction of serine 1177 phosphorylation in eNOS (Xia et al., 2016). We have also reported that the acetylation of eNOS in PVAT is enhanced in HFD-induced obesity model (Xia et al., 2017b). The treatment with WS^®^ 1442, a standardized *Crataegus* extract, leads to a complete restoration of vascular function in PVAT-containing aorta of HFD-fed mice and have no effects on body weight or fat mass (Xia et al., 2017b). WS^®^ 1442 reverses the reduced phosphorylation and enhanced acetylation of PVAT eNOS caused by HFD feeding. Interestingly, WS^®^ 1442 treatment does not affect SIRT1 expression but improves SIRT1 activity and function by increasing NAD^+^ production via nicotinamide phosphoribosyltransferase (NAMPT) (Xia et al., 2017b).

In low-density lipoprotein receptor knockout (LDLr-KO) mice, a model of human familial hypercholesterolemia, the presence of PVAT protects against impaired endothelium-dependent relaxation to acetylcholine and insulin. The thoracic aortic PVAT in LDLr-KO mice shows enhanced eNOS expression and NO levels, suggesting the protective role of PVAT in the familial hypercholesterolemia model by enhancing eNOS expression and improving endothelial function (Baltieri et al., 2018). However, Du et al. (2015) also suggested that PVAT-derived IL-6 promotes the pathogenesis of arterial stiffness and remodeling in LDLr-KO mice. These suggest that the PVAT-derived NO and adipokine regulation is essential for vascular function and could be a potential novel therapeutic target for vascular aging.

Recent studies have provided evidence that resveratrol improves PVAT function, suggesting the importance of SIRT1 in modulating PVAT function (Sun et al., 2014; Chen et al., 2016). Activation of the SIRT1/AMPK signaling in PVAT can beneficially regulate adipokine expression, ameliorate endothelial dysfunction caused by inhibiting NFκB activation, and alter PVAT inflammation induced by fructose- (Chen et al., 2016) or HFD-feeding (Sun et al., 2014). The oxidative stress in PVAT may lead to increased pro-inflammatory cytokine and chemokine secretion, and the superoxide derived from PVAT promotes artery stiffening in aged mice (Fleenor et al., 2014). Resveratrol or other anti-oxidants may improve PVAT function via scavenging the superoxide and normalize the expression of TNF−α, IL−6, MCP−1, adiponectin, PPARγ, and eNOS phosphorylation in PVAT (Chen et al., 2016). The above *ex vivo* experiments using conditional media derived from PVAT suggest that the observed effects of resveratrol are attributed solely to PVAT.

Nitric oxide released from PVAT contributes to the enhancement of vascular relaxation, as described by Gil-Ortega et al. (2010). Moreover, various recent studies have reported the gene and protein expression of eNOS in PVAT (Xia et al., 2016; Baltieri et al., 2018). HFD leads to the reduction of eNOS and NO production in PVAT (Gil-Ortega et al., 2009), while it is suggested to be mediated by leptin (Gil-Ortega et al., 2010). Despite the above evidence, eNOS expression is detected specifically in WAT (Ribiere et al., 1996) and adipocytes (Elizalde et al., 2000). In addition, the immunobiological staining suggests that the eNOS expression in PVAT is located in the endothelial cells of microvessels as well as the adipocytes (Dashwood et al., 2007). It is also suggested that the adipocyte-derived NO is released into the interstitial fluid and diffuse into the adjacent vessels (Mastronardi et al., 2002).

One of the mechanisms leading to PVAT dysfunction is the leptin and adiponectin dysregulation. As mentioned, HFD-induced enhanced leptin level in PVAT leads to the reduction of eNOS and NO production (Gil-Ortega et al., 2010). Moreover, plasma adiponectin levels and adiponectin expression in adipose tissue are decreased in eNOS knockout mice, and inactivation of eNOS decreases rosiglitazone-induced adiponectin secretion in cultured adipocytes (Koh et al., 2010). PVAT may secrete adiponectin, which is known to normalize endothelial function, partly by enhancing eNOS phosphorylation in the endothelium (Sena et al., 2017). Interestingly, SIRT1 is reported to regulate adiponectin secretion in adipocytes (Qiang et al., 2007), possibly via FOXO1 (Qiao and Shao, 2006). These findings also suggest that the SIRT1/FOXO axis is an important player in regulating eNOS and adiponectin in adipocytes.

The function of PVAT is determined by the browning and inflammation status, rather than by its size. The thermogenic properties of PVAT have been demonstrated as anti-atherogenic (Brown et al., 2014). Fitzgibbons et al. (2011) have suggested that the thoracic PVAT of mice shows a very low inflammation even after long-term HFD treatment, probably due to the high similarity with the phenotype of brown adipose tissue (BAT). These observations suggest an interesting hypothesis that promoting browning of PVAT might have a protective effect on the development of vascular diseases. However, the detailed mechanisms underlying browning or the thermogenesis of PVAT are poorly known. Nevertheless, mitochondrial biogenesis is important in adipocyte browning (Lemecha et al., 2018). Mitochondrial function is linked to adiponectin production in adipocytes, and enhanced mitochondrial function is needed for adipocyte differentiation (Koh et al., 2007). Recent studies suggest that eNOS-derived NO can promote mitochondrial biogenesis (Csiszar et al., 2009), indicating the vital role of eNOS in the adipocytes in adiponectin synthesis and mitochondrial biogenesis. In addition, eNOS is abundantly expressed in both BAT and isolated brown adipocytes (Kikuchi-Utsumi et al., 2002), suggesting that PVAT eNOS could also facilitate browning or the thermogenesis of PVAT. Therefore, in future studies, targeting SIRT1/eNOS-mediated PVAT adaptive thermogenesis may be helpful in elucidating its beneficial effects on endothelial function against vascular injury.

**FIGURE 3:**
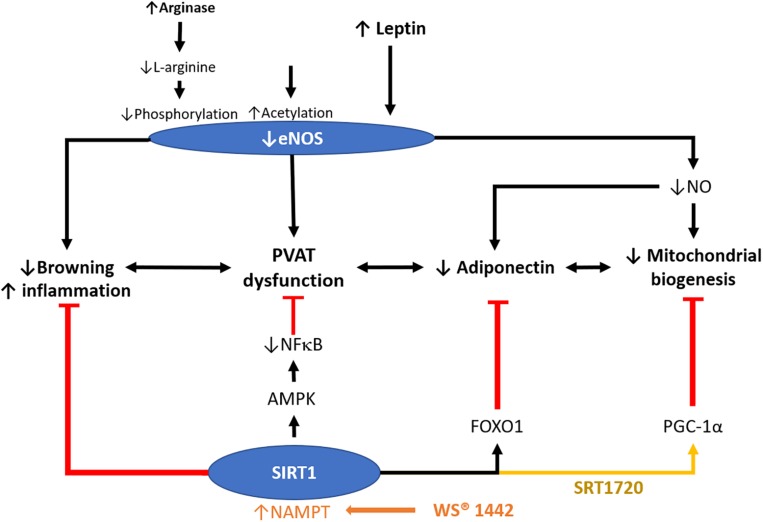
Role of SIRT1 in modulating PVAT function. PVAT is critical in modulating vascular function. *Crataegus* extract WS^®^ 1442 activates SIRT1 via increased NAD^+^ production by NAMPT. WS^®^ 1442 prevents PVAT dysfunction via reversing the reduced phosphorylation and enhanced acetylation of PVAT eNOS caused by HFD feeding. SIRT1 ameliorates PVAT dysfunction caused by NFκB activation and PVAT inflammation fructose (Chen et al., 2016) or HFD (Sun et al., 2014). SIRT1 also increases mitochondrial biogenesis via PGC-1α. NO, nitric oxide. HFD, high-fat diet. PGC-1α, peroxisome proliferator-activated receptor-gamma and coactivator-1 alpha. NF-κB, nuclear factor kappa-light-chain-enhancer of activated B cells. NAMPT, nicotinamide phosphoribosyltransferase. AMPK, 5′ AMP-activated protein kinase.

Adipose tissue-specific-SIRT1 deletion augments obesity-induced brown-to-white transition in PVAT *in vivo* and leads to impaired vascular reactivity. PVAT SIRT1 plays a pivotal role in controlling PVAT browning by reducing local superoxide production and enhancing adipokines production to protect from vascular injury (Gu et al., 2015). Activation of SIRT1 promotes recovery of mitochondrial protein and function by increasing mitochondrial biogenesis via the peroxisome proliferator-activated receptor-gamma and coactivator 1 alpha (PGC-1α) mitochondrial pathway in adipose tissue (Nemoto et al., 2005). SIRT1 specific activator SRT1720 treatment in obese mice could prolong the lifespan and reverse organ damages induced by HFD via normalized PGC-1α acetylation thereby improving mitochondrial biogenesis (Minor et al., 2011); however, there exists a lack of evidence dissecting the function of adipose SIRT1 in mediating these processes, and whether PGC-1α is involved in the protective role of SIRT1 in PVAT remain unidentified. It would, therefore, be interesting to investigate the critical role of the interplay between PVAT SIRT1 and eNOS in controlling the browning and inflammation status of PVAT that mediates vascular function. The molecular mechanisms on whether the SIRT1 function is regulated in PVAT through an eNOS-dependent or independent way, are of particular interest (Figure 3). Given the fact that NO regulates SIRT1 expression in WAT and white adipocytes (Nisoli et al., 2005), it is conceivable that NO may also regulate SIRT1 in PVAT. Elucidating the molecular mechanism on how SIRT1 and eNOS facilitate PVAT function and improve the partnership of PVAT SIRT1 and eNOS in mitochondrial biogenesis should be focused in future PVAT researches. Moreover, the research gap may also fall on whether there is a unique and specific function of adipose eNOS in modulating vascular functions.

## SIRT1 and Vascular Aging

“Senescence,” is derived from the Latin word “*senex*,” meaning aged man, and cellular senescence refers to the stress and damage response that causes cell growth arrest and impaired cellular function (Shelton et al., 1999). There are two types of cell senescence: replicative senescence and stress-induced premature senescence (Bai et al., 2014). The age-dependent structural or biological changes of the vascular system, including the endothelium and vascular smooth muscle cells, is referred to as the vascular aging. Endothelial senescence may result in pro-atherosclerotic, pro-inflammatory, and prothrombotic changes in vascular function and contributes to the age-related vascular disease (Erusalimsky, 2009; Bai and Wang, 2013).

SIRT1 is a potential therapeutic target for longevity and anti-aging in mammals. In senescent endothelial cells, SIRT1 protein expression and activity are impaired as a result of both transcriptional and post-translational modifications. Both SIRT1 expression and function decrease progressively in replicative senescent cultured endothelial cells (Zu et al., 2010), while decreased SIRT1 expression is also observed in cultured primary human vascular smooth muscle cells (Takemura et al., 2011). Dysfunction of SIRT1 or its reduced expression promotes endothelial senescence, which is accompanied by an increased LKB1 expression. Both LKB1 and AMPK-induced senescence in endothelial cells can be antagonized by overexpression of SIRT1 (Zu et al., 2010). These suggest that SIRT1 expression and function is negatively correlated to vascular aging.

SIRT1 prevents endothelial senescence by deacetylation of PGC-1α and PPARα activation, resulting in the reduction of both NADPH oxidase-mediated ROS production and NO inactivation (Zarzuelo et al., 2013). SIRT1 also elicits the anti-senescence functions in primary mouse embryonic fibroblasts by regulating p53 deacetylation (Langley et al., 2002). SIRT1 activation by resveratrol prevents oxidative stress-induced aging in endothelial cells by stimulating AMPK-mediated downstream cascade (Ido et al., 2015). SIRT1 also promotes mitochondrial function and reduces mitochondrial production of ROS (Handy and Loscalzo, 2012). In aged arteries, reduced SIRT1 expression leads to downregulation of soluble guanylyl cyclase (sGC), thus compromising vasodilator responses by a decreased NO-sGC-cGMP signaling. Overexpression of SIRT1 in the endothelium of aged arteries prevents age-induced alteration in arterial responses to phenylephrine or acetylcholine by enhancing notch signal transduction, which upregulates sGCβ expression (Guo et al., 2018). In contrast, pharmacological activation of SIRT1 by SRT1720 reversed the apoptotic endothelial cells and protected against endothelial senescence via eNOS induction (Li et al., 2016).

Senescent endothelial cells have reduced levels of NO production due to decreased eNOS expression and phosphorylation (Sato et al., 1993; Hoffmann et al., 2001; Matsushita et al., 2001). In aged arteries, the ability of the endothelium to promote vasodilation is significantly reduced due to the decreased eNOS expression, NO bioavailability, or sGC activity in the endothelium (Klöß et al., 2000; Csiszar et al., 2002). Retardation of cellular senescence by NO donors was first reported by Vasa et al. (2000). Activation of eNOS and/or increasing NO could delay endothelial senescence via the administration of NO boosting substances, such as L-arginine and L-citrulline in the cell model (Hayashi et al., 2006). NO donors are shown to inhibit age-related downregulation of telomerase activity and subsequent senescence in HUVECs (Vasa et al., 2000). A f3ew studies also suggest that NO suppresses ROS production in HUVECs (Hayashi et al., 2006; Selemidis et al., 2007), while the underlying mechanism on how NO reduces ROS production in preventing senescence remains unclear. As SIRT1 is a key player interacting with eNOS and NO, NO might suppress ROS production by stimulating SIRT1 in targeting endothelial senescence.

**FIGURE 4:**
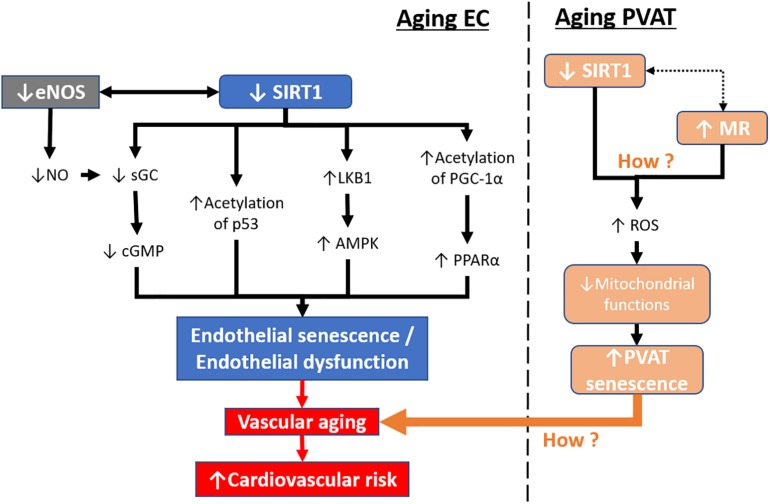
Aging-induced SIRT1 downregulation in endothelial cells and PVAT. During aging, SIRT1 expression and activity are reduced in senescent endothelial cells. Downstream targets of SIRT1, including sGC, p53, LKB1, and PGC-1α, are altered leading to endothelial senescence. In contrast, senescent endothelial cells have lower levels of eNOS activity and reduced levels of NO production. Mitochondrial dysfunction in PVAT can lead to PVAT senescence resulting in the loss of its anticontractile properties. The endothelial senescence, together with PVAT senescence, lead to vascular aging, causing increased cardiovascular risk. sGC, soluble guanylyl cyclase. cGMP, cyclic guanosine monophosphate. LKB1, liver kinase b1, AMPK, 5′ AMP-activated protein kinase. PGC-1α, peroxisome proliferator-activated receptor-gamma and coactivator-1 alpha. PPARα, peroxisome proliferator-activated receptor alpha. MR, mineralocorticoid receptor. ROS, reactive oxidative species.

Apart from endothelial senescence, senescence of other vascular tissues, i.e., smooth muscle or PVAT, may also lead to vascular aging. Endothelial senescence can lead to changes in protein expression that are associated with cellular architecture, cytoskeletal function, and extracellular matrix remodeling (Shelton et al., 1999; Vasile et al., 2001; Kamino et al., 2003; Chang et al., 2005). Endothelial senescence itself cannot completely explain the phenotype and progression of vascular aging. Recently, PVAT has received much attention in the field of vascular biology because of its effects in modulating vascular function by releasing various adipokines. Therefore, it is also possible that the senescence of PVAT contributes to vascular aging. To date, few studies have investigated PVAT senescence, and both aging and obesity might affect PVAT in a comparable manner (Miao and Li, 2012). A recent report shows that mineralocorticoid receptor (MR) is activated in the mitochondria of PVAT which causes premature-aging in adipose tissue and senescence in obese mice model, resulting in the loss of PVAT anticontractile properties (Lefranc et al., 2019). This suggests that mitochondrial dysfunction in PVAT may contribute to the aging phenotype, which is worth further investigation. SIRT1 is reported to be important in promoting mitochondrial function and reduces the production of ROS (Handy and Loscalzo, 2012). However, there are no reports on how SIRT1 regulates the senescence of PVAT. Therefore, the beneficial effect of SIRT in targeting mitochondrial dysfunction and aging-related processes in PVAT could be a promising future research direction in the treatment of vascular aging (Figure 4).

## Conclusion

Vascular function regulation relies on the interplay between endothelium and PVAT and the remodeling process in the vascular smooth muscles. SIRT1 in the endothelium, smooth muscle, and PVAT play a critical role in vasoprotection by regulating a myriad of signaling pathways. Previous studies have focused on endothelial SIRT1 and demonstrate the beneficial effects of SIRT1 in endothelial function. In the endothelium, there exists a dynamic regulation between SIRT1 and eNOS that is crucial for maintaining endothelial functions and vascular remodeling. Recent studies have shifted the focus to investigate SIRT1 function in PVAT and arterial remodeling. PVAT has been understood to be an essential and active component of the vascular system and promoting browning of PVAT might have protective effects on the development of vascular diseases. eNOS expression in the adipocyte is poorly understood, yet a vital key for the PVAT function. SIRT1 and eNOS partnership in modulating the PVAT phenotype and adipokines secretion could be an important field of research; however, the underlying mechanisms of the interplay between SIRT1 and eNOS in arterial remodeling, vascular aging, and PVAT function have not been completely understood and require further studies. Understanding the molecular mechanism is crucial for the development of novel therapy to treat metabolic disease-induced vascular complications. Further research on the endothelium, smooth muscle, and PVAT could, therefore, enhance the understanding of the pathogenesis of vascular aging. Several studies have described the beneficial effects of SIRT1 in endothelial function; in addition, SIRT1 is a potential therapeutic target in reversing abnormal arterial remodeling and PVAT dysfunction.

## Author Contributions

AM wrote the initial draft of the manuscript. HL and NX critically reviewed and edited the manuscript. All authors agreed to its publication.

## Conflict of Interest Statement

The authors declare that the research was conducted in the absence of any commercial or financial relationships that could be construed as a potential conflict of interest.
